# Neoadjuvant Chemotherapy is Associated with Worse 5-Year Overall Survival in Patients with Metaplastic Breast Cancer Compared with Primary Surgery: A National Cancer Database Analysis

**DOI:** 10.1245/s10434-025-18085-z

**Published:** 2025-08-20

**Authors:** Anna M. Chichura, Xiaoying Chen, Margo Nelis, Lauren Kopicky, Wei Wei, Azka Ali, Rahul Tendulkar, Zahraa Al-Hilli

**Affiliations:** 1 Division of Surgical Oncology, Department of Surgery, University of Illinois at Chicago, Chicago, IL USA; 2 Division of Gynecologic Oncology, Department of Obstetrics and Gynecology, University of Illinois at Chicago, Chicago, IL USA; 3 Department of Quantitative Health Sciences, Cleveland Clinic, Cleveland, OH USA; 4 Obstetrics and Gynecology Institute, Cleveland Clinic, Cleveland, OH USA; 5 Breast Center, Integrated Surgical Institute, Cleveland Clinic, Cleveland, OH USA; 6 Department of Medical Oncology, Taussig Cancer Institute, Cleveland Clinic, Cleveland, OH USA; 7 Department of Radiation Oncology, Taussig Cancer Institute, Cleveland Clinic, Cleveland, OH USA

## Abstract

**Background:**

Metaplastic breast carcinoma (MpBC) is a rare breast cancer subtype frequently exhibiting triple-negative receptor status and poor response to chemotherapy. The optimal treatment sequence for MpBC remains unclear. This study evaluated overall survival (OS) in MpBC patients treated with neoadjuvant chemotherapy (NACT) versus upfront surgery and adjuvant chemotherapy using the National Cancer Database (NCDB).

**Methods:**

A retrospective cohort analysis was performed using the NCDB to identify patients with stage I–III MpBC diagnosed from 2010 to 2019 who received either NACT followed by surgery or upfront surgery and adjuvant chemotherapy. Patients with metastatic disease or missing survival data were excluded. Kaplan–Meier and Cox proportional hazards models were used to estimate OS. Primary endpoints included pathologic complete response (pCR), breast conservation rate, and 5-year OS.

**Results:**

Of 5136 patients, 3491 (67.9%) underwent upfront surgery and 1645 (32.1%) received NACT. The 5-year OS was significantly better in patients treated with upfront surgery and adjuvant chemotherapy (hazard ratio [HR] 0.556, 95% confidence interval [CI] 0.464–0.667; *p* < 0.0001). The pCR rate was 4.92%. Patients receiving NACT had a lower breast conservation rate than those undergoing upfront surgery (33.25% vs. 49.53%; *p* < 0.0001). Adjuvant chest wall radiation was associated with improved OS (HR 0.641, 95% CI 0.536–0.766, *p* < 0.0001).

**Conclusions:**

NACT was associated with worse OS in MpBC, likely due to its chemotherapy-resistant biology and low pCR rate. For patients with resectable MpBC, upfront surgery and adjuvant chemotherapy and radiation may offer better outcomes. Further research is needed to clarify the role of immunotherapy and advance systemic treatment strategies.

**Supplementary Information:**

The online version contains supplementary material available at 10.1245/s10434-025-18085-z.

Metaplastic breast carcinoma (MpBC) is a rare morphological subtype of breast cancer, representing 0.2–5% of invasive cases.^1^ Historically, over 70% of MpBC cases are classified as triple-negative breast cancer (TNBC) and therefore lack clinically targetable biomarkers such as estrogen receptor (ER), progesterone receptor (PR), or human epidermal growth factor receptor 2 (HER2).^6^ As a result, cytotoxic chemotherapy is often the primary systemic therapeutic option available. Metaplastic TNBC generally has a worse prognosis compared with other morphological subtypes of TNBC and shows a poorer response to neoadjuvant chemotherapy (NACT).^7,8^

Current National Comprehensive Cancer Network (NCCN) guidelines do not make distinct recommendations for aggressive subsets such as MpBC, and neoadjuvant systemic treatment remains the standard of care for patients presenting with locally advanced disease.^2^ However, it is not known if there is an advantage to NACT over upfront surgery for this distinct subset of tumors. This study aimed to assess the clinical outcomes in patients with MpBC treated with neoadjuvant systemic therapy versus upfront surgery followed by adjuvant chemotherapy.

## Methods

A retrospective analysis was performed using the National Cancer Database (NCDB), a publicly available database composed of de-identified information collected from over 70% of newly diagnosed cancer cases at any of the 1500 Commission on Cancer-accredited facilities in the United States (US).^12^ The NCDB was reviewed for patients with stage I–III MpBC diagnosed from 2010 to 2019 who were treated with upfront surgery followed by adjuvant chemotherapy or neoadjuvant systemic therapy followed by surgical resection. Patients with metastatic (stage IV) or non-invasive (stage 0) disease, absent or unknown tumor size, a surgical procedure coded as ‘none’, ‘local tumor destruction only’, ‘not otherwise specified’, or ‘unknown’ were excluded.Patients with unknown or missing survival data were excluded from the survival analysis. Clinicopathologic data were abstracted, including clinical and pathologic T and N categories; ER, PR, and HER2 status; use of neoadjuvant chemotherapeutic agents; use of neoadjuvant immunotherapeutic agents; rate of upfront surgery; type of surgery performed; use of adjuvant chemotherapy; and use of adjuvant radiation. Primary study endpoints included pathologic complete response (pCR) to NACT, rate of breast conservation, and overall survival (OS).

Patient characteristics were summarized using descriptive statistics. Continuous variables were reported as median with interquartile range (IQR) and range, while categorical variables were presented as frequencies and percentages. Treatment group comparisons for continuous variables were conducted using the Wilcoxon rank-sum test. Categorical variables were compared using the Chi-square test as well as primary endpoints of complete response and rate of breast conservation. OS was analyzed using the Kaplan–Meier method and differences between treatment groups were assessed using the log-rank test. Multivariable analyses were performed using Cox proportional hazards models for survival outcomes, adjusting for clinically relevant covariates. All statistical tests were two-sided and a *p*-value < 0.05 was considered statistically significant. Statistical analysis was carried out using SAS Studio 3.7 (SAS Institute, Cary, NC, USA) and R version 4.2 (R Foundation for Statistical Computing, Vienna, Austria).

## Results

**Fig. 1:**
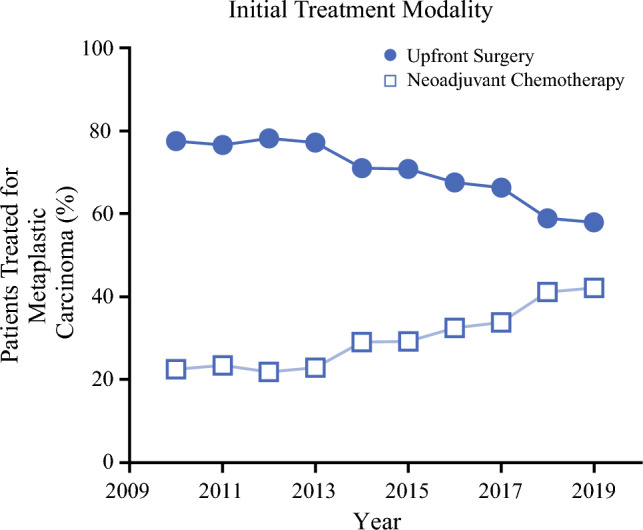
Proportion of patients treated for metaplastic breast cancer undergoing upfront surgery or neoadjuvant chemotherapy between 2010 and 2019

Between 2010 and 2019, a total of 5136 patients were diagnosed with MpBC, with a median follow up of 63.44 months (range 2.3–144.89). Of these patients, 3491 (67.9%) underwent upfront surgery and 1645 (32.1%) received NACT. During this time, there was a statistically significant shift in initial treatment strategy among breast cancer patients (*p* < 0.0001) (Table 1). The proportion of patients undergoing systemic therapy prior to surgery increased from 22.5% in 2010 to 42.1% in 2019. In contrast, the proportion of patients treated with surgery first decreased from 77.5% to 57.9% over the same period (Fig. 1).

**Table 1:** Demographic and clinicopathologic characteristics of patients with metaplastic breast cancer undergoing upfront surgery or receiving neoadjuvant chemotherapy, National Cancer Database, 2010–2019

	Upfront surgery	Neoadjuvant chemotherapy	All	*p*-Value
All	3491 (100)	1645 (100)	5136 (100)	
Age, years				< 0.0001
< 50	661 (18.93)	484 (29.42)	1145 (22.29)	
50–69	1947 (55.77)	905 (55.02)	2852 (55.53)	
≥ 70	883 (25.29)	256 (15.56)	1139 (22.18)	
Sex			0.1617	
Female	3476 (99.57)	1642 (99.82)	5118 (99.65)	
Male	15 (0.43)	3 (0.18)	18 (0.35)	
Year of diagnosis				< 0.0001
2010	258 (7.39)	75 (4.56)	333 (6.48)	
2011	336 (9.62)	103 (6.26)	439 (8.55)	
2012	357 (10.23)	100 (6.08)	457 (8.9)	
2013	360 (10.31)	107 (6.5)	467 (9.09)	
2014	339 (9.71)	139 (8.45)	478 (9.31)	
2015	346 (9.91)	143 (8.69)	489 (9.52)	
2016	345 (9.88)	166 (10.09)	511 (9.95)	
2017	384 (11)	196 (11.91)	580 (11.29)	
2018	259 (7.42)	181 (11)	440 (8.57)	
2019	264 (7.56)	192 (11.67)	456 (8.88)	
Race				0.002
African American	618 (17.7)	359 (21.82)	977 (19.02)	
Asian	168 (4.81)	74 (4.5)	242 (4.71)	
Caucasian	2688 (77)	1203 (73.13)	3891 (75.76)	
Other/unknown	17 (0.49)	9 (0.55)	26 (051)	
Insurance			< 0.0001	
Government	1653 (2.98)	676 (41.09)	2329 (45.35)	
Private	1734 (49.67)	905 (55.02)	2639 (51.38)	
Other/unknown	104 (2.98)	64 (3.89)	168 (3.27)	
Facility type				< 0.0001
Community cancer program	214 (6.13)	74 (4.5)	288 (5.61)	
Comprehensive community cancer program	1314 (37.64)	488 (29.67)	1802 (35.09)	
Academic/research program	1110 (31.8)	530 (32.22)	1640 (31.93)	
Integrated network cancer program	688 (19.71)	374 (22.74)	1062 (20.68)	
Other/unknown	165 (4.73)	179 (10.88)	344 (6.7)	
Charlson–Deyo score				0.0067
0	2795 (80.06)	1381 (83.95)	4176 (81.31)	
1	517 (14.81)	193 (11.73)	710 (13.82)	
2	122 (3.49)	53 (3.22)	175 (3.41)	
3	57 (1.63)	18 (1.09)	75 (1.46)	
Grade				< 0.0001
1	54 (1.55)	6 (0.36)	60 (1.17)	
2	332 (9.51)	79 (4.8)	411 (8)	
3	2006 (57.46)	803 (48.81)	2809 (54.69)	
Unknown	1099 (31.48)	757 (46.02)	1856 (36.14)	
Receptor status				< 0.0001
HR+/HER2−	623 (17.85)	178 (10.82)	801 (15.6)	
HER2+	68 (1.95)	73 (4.44)	141 (2.75)	
Triple-negative	1952 (55.92)	747 (45.41)	2699 (52.55)	
Unknown	848 (24.29)	647 (39.33)	1495 (29.11)	
Surgery				< 0.0001
Lumpectomy	1729 (49.53)	547 (33.25)	2276 (44.31)	
Mastectomy	1762 (50.47)	1098 (66.75)	2860 (55.69)	
Radiation				< 0.0001
Breast/chest wall	2067 (59.21)	1102 (66.99)	3169 (61.7)	
None	1335 (38.24)	498 (30.27)	1833 (35.69)	
Unknown	89 (2.55)	45 (2.74)	134 (2.61)	
*Clinical staging*				
cT				< 0.0001
1	1401 (40.13)	178 (10.82)	1579 (30.74)	
2	1687 (48.32)	879 (53.43)	2566 (49.96)	
3	296 (8.48)	386 (23.47)	682 (13.28)	
4	90 (2.58)	198 (12.04)	288 (5.61)	
Unknown	17 (0.49)	4 (0.24)	21 (0.41)	
cN				< 0.0001
0	3142 (90)	1080 (65.65)	4222 (82.2)	
1	269 (7.71)	421 (25.59)	690 (13.43)	
2	38 (1.09)	85 (5.17)	123 (2.39)	
3	13 (0.37)	53 (3.22)	66 (1.29)	
Unknown	29 (0.83)	6 (0.36)	35 (0.68)	
Clinical stage				< 0.0001
1	1372 (39.3)	146 (8.88)	1518 (29.56)	
2	1888 (54.08)	967 (58.78)	2855 (55.59)	
3	230 (6.59)	532 (32.34)	762 (14.84)	
Unknown	1 (0.03)	0 (0)	1 (0.02)	
*Pathologic staging*				
pT				< 0.0001
0	5 (0.14)	41 (2.49)	46 (0.9)	
is	1 (0.03)	13 (0.79)	14 (0.27)	
1	1171 (33.54)	351 (21.34)	1522 (29.63)	
2	1783 (51.07)	352 (21.4)	2135 (41.57)	
3	374 (10.71)	226 (13.74)	600 (11.68)	
4	94 (2.69)	97 (5.9)	191 (3.72)	
Unknown	63 (1.8)	565 (34.35)	628 (12.23)	
pN				< 0.0001
0	2788 (79.86)	764 (46..44)	3552 (69.16)	
1	445 (12.75)	213 (12.95)	658 (12.81)	
2	90 (2.58)	66 (4.01)	156 (3.04)	
3	34 (0.97)	20 (1.22)	54 (1.05)	
Unknown	134 (3.84)	582 (35.38)	716 (13.94)	
Pathologic stage				< 0.0001
0	3 (0.09)	36 (2.19)	39 (0.76)	
1	1131 (32.4)	300 (18.24)	1431 (27.86)	
2	1961 (56.17)	508 (30.88)	2469 (48.07)	
3	327 (9.37)	231 (14.04)	558 (10.86)	
Unknown	69 (1.98)	570 (34.65)	639 (12.44)	
Pathologic complete response				
Yes	4 (0.11)	81 (4.92)	85 (1.65)	< 0.0001
No	3487 (99.89)	1564 (95.08)	5051 (98.35)	

Data are expressed as *n* (%)

*HR* hormone receptor, *HER2* human epidermal growth factor receptor 2

Univariate analysis revealed several clinicopathologic differences between MpBC patients undergoing upfront surgery and those receiving NACT (Table 1). Most patients were under the age of 70 years at the time of diagnosis (*n* = 3997, 77.8%) and had triple-negative disease (*n* = 2699, 52.55%). Patients receiving NACT were more often younger (*p* < 0.001), had a higher proportion of HER2+ receptor status (*p* < 0.001), had a higher proportion of clinical (cN) nodal involvement (*p* < 0.001), underwent mastectomy at higher proportions (66.75% vs. 50.47%; *p* < 0.0001), and received breast and chest wall irradiation at higher proportions (66.99% vs. 59.21%; *p* < 0.0001). pCR was observed in 81 patients (4.92%). Patients undergoing upfront surgery had higher proportions of hormone receptor-positive (HR+)/HER2− and triple-negative receptor status (*p* < 0.001), had lower clinical (cT) [*p* < 0.0001] and pathologic (pT) tumor stage (*p* < 0.0001) and less frequent nodal involvement (pathologic N [pN] stage 0 disease) [*p* < 0.0001], and underwent lumpectomy at higher proportions (49.53% vs. 33.25%; *p* < 0.0001).

Univariate analysis also revealed several demographic differences between MpBC patients undergoing upfront surgery and those receiving NACT (Table 1). The group of patients undergoing upfront surgery included a higher proportion of Caucasian patients (77% vs. 73.13%; *p* = 0.002) and were more likely to be treated at a Comprehensive Community Cancer Program (37.64% vs. 29.67%; *p* < 0.0001). The group of patients who received NACT included a higher proportion of non-Hispanic Black patients (21.82% vs. 17.7%; *p* = 0.002), a higher proportion of patients with a Charlson–Deyo (CD) comorbidity score of 0 (83.95% vs. 80.06%; *p* = 0.0067), and were more likely to use private insurance (55.02% vs. 49.67%; *p* < 0.0001).

**Fig. 2:**
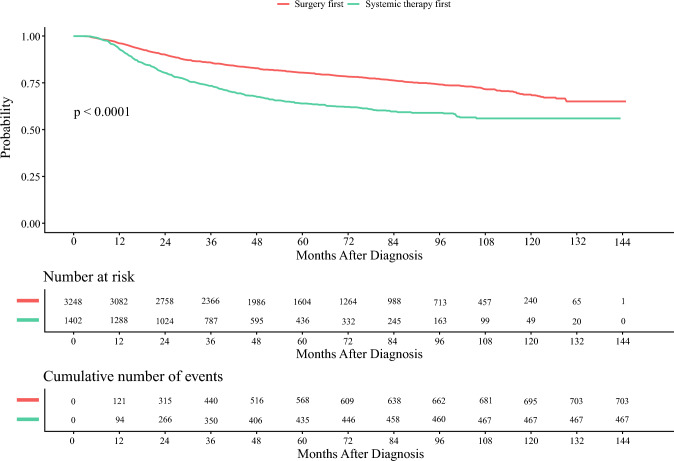
Adjusted overall survival of patients with metaplastic breast cancer undergoing upfront surgery or receiving neoadjuvant chemotherapy, National Cancer Database, 2010–2019. *Overall survival data were unavailable for 486 patients. Survival analysis was conducted on 4650 patients with available overall survival time and event data

The 5-year OS of patients was 76% (95% confidence interval [CI] 0.74–0.77) [electronic supplementary material (ESM) Table 1]. The median OS was not reached. Patients undergoing upfront surgery followed by adjuvant chemotherapy had an increased unadjusted 5-year OS compared with patients receiving NAC (ESM Table 1), and this survival difference persisted in multivariate analysis (hazard ratio [HR] 0.556, 95% CI 0.464–0.667, *p* < 0.0001) (Fig. 2, Table 2). Higher Charlson–Deyo comorbidity score (HR 1.913, 95% CI 1.127–3.248, *p* = 0.0163), pathologic tumor stage (pT3: HR 7.447, 95% CI 1.558–35.604, *p* = 0.0119; pT4: HR 15.199, 95% CI 3.048–75.798, *p* = 0.0009), and higher pathologic nodal stage (pN1: HR 2.156, 95% CI 1.704–2.728; pN2: HR 2.598, 95% CI 1.719–3.926; pN3: HR 3.928, 95% CI 2.222–6.944, all *p* < 0.0001) were associated with worse 5-year OS (Table 2). Age  ≤ 70 years (age < 50 years: HR 0.684, 95% CI 0.526–0.889, *p* = 0.0045; age 50–69 years: HR 0.723, 95% CI 0.598–0.875, *p* = 0.0009), and adjuvant breast and chest wall radiation (HR 0.641, 95% CI 0.536–0.766, *p* < 0.0001) were associated with improved 5-year OS. No significant survival differences were seen according to sex, race, facility treatment type, tumor grade, receptor status, surgical approach, or clinical stage.

**Table 2:** Adjusted overall survival of patients with metaplastic breast cancer undergoing upfront surgery or receiving neoadjuvant chemotherapy, National Cancer Database, 2010–2019^a^

	Hazard ratio (95% CI)	*p*-value
Treatment sequence		
Neoadjuvant chemotherapy	Ref	
Upfront surgery	0.556 (0.464–0.667)	< 0.0001
Age, years		
< 50	0.684 (0.526–0.889)	0.0045
50–69	0.723 (0.598–0.875)	0.0009
≥70	Ref	
Sex		
Female	Ref	
Male	0.718 (0.177–2.911)	0.643
Race		
White	Ref	
African American	1.003 (0.83–1.213)	0.976
Asian	0.971 (0.648–1.454)	0.8858
Facility type		
Integrated network	Ref	
Community cancer program	0.954 (0.665–1.369)	0.7977
Comprehensive community cancer program	1.147 (0.938–1.403)	0.1809
Academic/research center	0.937 (0.76–1.155)	0.5428
Primary payor		
Private	Ref	
Government	1.255 (1.054–1.493)	0.0107
Charleson–Deyo		
0	Ref	
1	1.189 (0.976–1.449)	0.086
2	1.041 (0.699–1.55)	0.8437
3	1.913 (1.127–3.248)	0.0163
Grade		
1	Ref	
2	1.024 (0.52–2.018)	0.9449
3	1.029 (0.538–1.972)	0.9302
Receptor status		
Triple-negative	Ref	
HR+/HER2−	1.031 (0.859–1.237)	0.7414
HER2+	0.673 (0.433–1.045)	0.0775
Surgery		
Mastectomy	Ref	
Lumpectomy	1.091 (0.893–1.335)	0.3935
Radiation therapy		
None	Ref	
Breast/chest wall irradiation	0.641 (0.536–0.766)	< 0.0001
*Clinical staging*		
cT		
1	1.256 (0.599–2.636)	0.5467
2	1.49 (1.026–2.163)	0.8493
3	1.49 (1.026–2.163)	0.0362
4	Ref	
cN		
0	1.423 (0.775–2.613)	0.2551
1	1.087 (0.615–1.92)	0.7747
2	1.189 (0.64–2.207)	0.5835
3	Ref	
Clinical stage		
1	Ref	
2	1.252 (0.676–2.32)	0.4743
3	1.543 (0.727–3.276)	0.2589
*Pathologic staging*		
pT		
0	Ref	
is	4.229 (0.519–34.458)	0.178
1	2.489 (0.531–11.675)	0.2474
2	4.352 (0.925–20.475)	0.0627
3	7.447 (1.558–35.604)	0.0119
4	15.199 (3.048–75.798)	0.0009
pN		
0	Ref	
1	2.156 (1.704–2.728)	< 0.0001
2	2.598 (1.719–3.926)	0.0001
3	3.928 (2.222–6.944)	< 0.0001
Pathologic stage		
0	Ref	
1	1.67 (0.229–12.176)	0.6128
2	1.668 (0.231–12.032)	0.6119
3	1.617 (0.214–12.236)	0.6415

*CI* confidence interval, *HR* hormone receptor, *HER2* human epidermal growth factor receptor 2

^a^Overall survival data were unavailable for 486 patients. Survival analysis was conducted on 4650 patients with available overall survival time and event data

## Discussion

In the current study, we demonstrated that NACT was associated with worse OS in MpBC as compared with upfront surgery followed by adjuvant chemotherapy. The pCR rate was low and there was no increase in the rate of breast conservation following NACT. In contrast, radiation therapy was associated with improved OS.

MpBC is a rare and aggressive subtype of breast cancer. In a prior study from the NCDB of patients diagnosed with MpBC between 2010 and 2014, those with MpBC were found to have a significantly lower 5-year OS rate of 72.7% compared with 87.5% among those with non-MpBC.^3^ MpBC has also been shown to have a worse 5-year OS compared with non-metaplastic subtype TNBC.^3,4^

The current World Health Organization (WHO) classification of metaplastic carcinoma includes adenosquamous carcinoma, squamous cell carcinoma, spindle cell carcinoma, fibromatosis-like metaplastic carcinoma, and metaplastic carcinoma with mesenchymal differentiation (e.g. chondroid, osseous, rhabdomyoid, or neuroglial elements).^5^ Only low-grade adenosquamous and low-grade fibromatosis-like carcinoma are considered to have a favorable prognosis without adjuvant systemic therapies.^2^ Of the other subtypes, metaplastic carcinoma with mesenchymal differentiation appears to have the best 5-year breast cancer-specific survival (BCSS), and squamous MpBC the worst.^6-8^

Over 70% of MpBC cases are classified as TNBC, making cytotoxic chemotherapy the primary systemic treatment option.^2,3,9^ However, despite their triple-negative receptor status, MpBC patients historically have a poor response to chemotherapy, with anywhere from 28 to 46.2% of patients progressing on initial treatment, and some studies suggesting no benefit to chemotherapy at all.^6,8,10-13^ Previous studies have also shown a relatively low pCR rate for MpBC ranging from 9.8 to 29%.^11-15^ Our study found a 4.92% pCR rate in this group of patients, which is notably lower than the expected pCR rate of TNBC to traditional NACT (51.2%) or the current standard of care, neoadjuvant chemoimmunotherapy (64.8%), as demonstrated in the KEYNOTE-522 study.^16^ However, the KEYNOTE-522 study did not specifically evaluate patients with metaplastic TNBC, and data specifically comparing neoadjuvant chemoimmunotherapy with standard NACT in metaplastic TNBC are limited. Nonetheless, regardless of chemotherapeutic regimen, achieving a pCR is associated with improved 5-year OS in both MpBC and other major subtypes of breast cancer.^13,17^ This highlights the importance of identifying patients who may respond to NACT, or developing novel treatment regimens that can improve response rate.

In addition to prognostic information, NACT can also offer the opportunity to de-escalate *or* escalate systemic therapy in the adjuvant setting. A higher residual cancer burden (RCB) score, which serves as a measure of residual cancer, has been associated with worse event-free survival for all breast cancer types, particularly in TNBC.^18^ Patients with stage I–III TNBC breast cancer treated with NACT with residual disease have been shown to have an improvement in disease-free survival (69.8% vs. 56.1%; HR 0.58, 95% CI 0.39–0.87) when treated with adjuvant capecitabine.^19^ Additionally, those with residual disease after NACT with germline *BRCA1* or *BRCA2* mutations have been shown to have an improvement in 3-year disease-free survival (87.5% vs. 80.4%; 95% CI 3.0–11.1) with adjuvant olaparib, once again suggesting that the presence of residual disease is a poor prognostic feature and adjuvant systemic therapy does improve outcomes in these otherwise high-risk patients.^20^

Another benefit of NACT is the potential to de-escalate surgery. Our study found that patients receiving NACT underwent mastectomy at a *higher* rate than patients undergoing upfront surgery (66.75% vs. 50.47%; *p* < 0.0001). However, regardless of whether patients underwent mastectomy or lumpectomy, surgical approach did not impact OS in our study. This is consistent with previously published data showing no difference in outcomes between breast conservation and mastectomy for MpBC.^3,21^

Our study did find improved OS for patients receiving adjuvant breast and chest wall irradiation, a finding consistent with existing literature. A study by Wang et al. utilized the Surveillance, Epidemiology, and End Results (SEER) database to investigate the impact of post-mastectomy radiotherapy (PMRT) on survival outcomes of patients with MpBC treated between 2010 and 2014.^22^ PMRT was found to significantly enhance the BCSS for patients with both intermediate-risk (T1-2, N1, M0) and high-risk (T1-2, N2-3, M0) disease. A novel machine learning model was recently developed to predict the survival rate for patients with MpBC.^23^ This model also demonstrated a survival benefit from radiation therapy in patients with stage I–III MpBC undergoing breast-conserving surgery as well as those undergoing mastectomy with T3-4/N2-3M0 disease.

Treatment sequence had not been extensively studied in MpBC prior to the current study. A 2021 study of 91 patients from a single institution investigated treatment sequence in 91 patients with MpBC, 60 of whom received NACT. The study did not find a significant difference in OS between receipt of NACT versus adjuvant chemotherapy, consistent with findings of the landmark National Surgical Adjuvant Breast and Bowel Project (NSABP) B-18 study.^24,25^ In the current study, patients receiving NACT had a worse 5-year OS, regardless of race, receptor subtype, or other prognostic factors historically associated with worse BCSS outcomes.^26^ This is likely in large part due to the chemoresistant nature of MpBC evidenced by the low response rate and a higher risk of progression while receiving NACT.

This study presents certain limitations in its broader applicability to the treatment of MpBC. In addition to the inherent limitations of a retrospective analysis of the NCDB, including the inability to specifically assess disease-specific survival, it lacks the capacity to examine the distinct subtypes of MpBC that may benefit from different treatment approaches. In particular, these data may not apply to the small subset of patients with HER2+ MpBC who would likely still benefit from standard-of-care HER2-targeted therapy in the neoadjuvant and adjuvant settings.^27^ It is also limited by its ability to evaluate survival in response to neoadjuvant chemoimmunotherapy, which only became standard of care after publication of the KEYNOTE-522 study in 2020 and its US FDA approval in July 2021.^16^ The potential application of immunotherapy in mTNBC is supported by the high expression of programmed death-ligand 1 (PD-L1)-positive tumor cells and the presence of intratumoral FOXP3+ regulatory T cells observed in metaplastic TNBC tumors, which may render them more susceptible to immune-based treatments and improve the response rate to neoadjuvant systemic therapy.^28^ Following this rationale, SWOG S1609 (DART: Dual Anti-CTLA-4 and Anti-PD-1 blockade in rare tumors) prospectively enrolled 17 patients with metastatic MpBC and treated them with the dual immune checkpoint inhibitors (ICI) nivolumab and ipilimumab. The trial met its primary endpoint of objective response rate (ORR), with a demonstrated ORR of 18%. Three of the 17 patients with MpBC achieved an objective response, with one complete response and two partial responses.^29^ Future prospective studies specifically evaluating the response of metaplastic TNBC to neoadjuvant chemoimmunotherapy, as well as the unique treatment needs of the various subtypes of MpBC and incorporating molecular profiling, are needed.^30^ Based on the data reviewed and available, we recommend that upfront surgery for MpBC leads to superior outcomes, particularly versus NACT, however it is yet to be seen if neoadjuvant chemoimmunotherapy or other novel neoadjuvant approaches in MpBC can significantly improve the otherwise low pCR rate. More data are desperately needed to answer these outstanding questions.

Despite its limitations, this study represents the largest contemporary analysis of treatment sequencing MpBC. The large sample size enabled robust multivariable modeling and subgroup analysis. Critically, this study fills a gap in the literature by directly comparing NACT with upfront surgery in MpBC.

## Conclusions

Patients with MpBC who underwent upfront surgery followed by adjuvant chemotherapy had improved OS compared with patients receiving NACT. For patients with resectable MpBC, upfront surgery followed by adjuvant chemotherapy and radiation may offer better outcomes. Further research is needed to clarify the role of immunotherapy in the treatment of MpBC and to identify patients most likely to benefit from systemic therapy. Continued advances in systemic treatment strategies are essential to inform optimal management.

## Supplementary Information

Below is the link to the electronic supplementary material.

Supplementary file1 (DOCX 23 kb)
