# Supplementary material for: Neoadjuvant Chemotherapy is Associated with Worse 5-Year Overall Survival in Patients with Metaplastic Breast Cancer Compared with Primary Surgery: A National Cancer Database Analysis
Source: Ann Surg Oncol. 2025 Aug 20;32(11):8525–33. doi: 10.1245/s10434-025-18085-z (PMC12494608; doi:10.1245/s10434-025-18085-z)
Supplement: Supplementary file 1 — Supplementary file1 (DOCX 23 kb) [file 10434_2025_18085_MOESM1_ESM.docx]

**Supplemental Table 1.** Unadjusted Overall Survival of Patients with Metaplastic Breast Cancer Undergoing Upfront Surgery or Receiving Neoadjuvant Chemotherapy, National Cancer Database, 2010-2019*

|  | | Total N | Number of Events | Median Survival (95%CI) | Rate at 5 Years (95%CI) | P-value |
| --- | --- | --- | --- | --- | --- | --- |
| All patients | | 4650 | 1170 | NA (NA, NA) | 0.76 (0.74, 0.77) |  |
| Treatment sequence | |  |  |  |  | < 0.0001 |
|  | Upfront surgery | 3248 | 703 | NA (NA, NA) | 0.81 (0.79, 0.82) |  |
|  | NACT | 1402 | 467 | NA (NA, NA) | 0.64 (0.61, 0.67) |  |
| Year of Diagnosis | |  |  |  |  | 0.2851 |
|  | 2010 | 333 | 108 | NA (NA, NA) | 0.78 (0.73, 0.82) |  |
|  | 2011 | 439 | 143 | NA (NA, NA) | 0.76 (0.72, 0.8) |  |
|  | 2012 | 457 | 128 | NA (NA, NA) | 0.79 (0.75, 0.83) |  |
|  | 2013 | 467 | 128 | NA (NA, NA) | 0.77 (0.73, 0.81) |  |
|  | 2014 | 478 | 147 | NA (NA, NA) | 0.74 (0.7, 0.78) |  |
|  | 2015 | 489 | 130 | NA (84.14, NA) | 0.75 (0.71, 0.79) |  |
|  | 2016 | 511 | 129 | NA (NA, NA) | 0.73 (0.69, 0.78) |  |
|  | 2017 | 580 | 111 | NA (NA, NA) | 0.79 (0.76, 0.83) |  |
|  | 2018 | 440 | 86 | NA (NA, NA) | NA |  |
|  | 2019 | 456 | 60 | NA (NA, NA) | NA |  |
| Age at diagnosis | |  |  |  |  | < 0.0001 |
|  | <50 | 1045 | 220 | NA (NA, NA) | 0.79 (0.76, 0.81) |  |
|  | 50-69 | 2583 | 591 | NA (NA, NA) | 0.78 (0.76, 0.8) |  |
|  | >=70 | 1022 | 359 | 110.88 (101.22, NA) | 0.67 (0.63, 0.7) |  |
| Sex | |  |  |  |  | 0.7882 |
|  | Female | 4632 | 1166 | NA (NA, NA) | 0.76 (0.74, 0.77) |  |
|  | Male | 18 | 4 | NA (93.01, NA) | 0.81 (0.63, 1) |  |
| Race | |  |  |  |  | 0.1066 |
|  | African American | 887 | 243 | NA (NA, NA) | 0.74 (0.71, 0.77) |  |
|  | Asian | 222 | 43 | NA (NA, NA) | 0.79 (0.73, 0.85) |  |
|  | Caucasian | 3519 | 876 | NA (NA, NA) | 0.76 (0.75, 0.78) |  |
| Facility Type | |  |  |  |  | 0.3095 |
|  | Community Cancer Program | 260 | 61 | NA (NA, NA) | 0.78 (0.72, 0.83) |  |
|  | Comprehensive Community Cancer Program | 1620 | 439 | NA (NA, NA) | 0.74 (0.72, 0.76) |  |
|  | Academic/ Research Program | 1495 | 365 | NA (NA, NA) | 0.77 (0.74, 0.79) |  |
|  | Integrated Network Cancer Program | 973 | 246 | NA (NA, NA) | 0.76 (0.73, 0.79) |  |
| Insurance | |  |  |  |  | < 0.0001 |
|  | Government | 2072 | 650 | NA (122.41, NA) | 0.7 (0.68, 0.72) |  |
|  | Private | 2426 | 483 | NA (NA, NA) | 0.81 (0.79, 0.83) |  |
| Charlson-Deyo Score | |  |  |  |  | < 0.0001 |
|  | 0 | 3789 | 882 | NA (NA, NA) | 0.77 (0.76, 0.79) |  |
|  | 1 | 641 | 209 | NA (115.29, NA) | 0.7 (0.66, 0.74) |  |
|  | 2 | 154 | 52 | 120.02 (78.55, NA) | 0.71 (0.64, 0.8) |  |
|  | 3 | 66 | 27 | 77.37 (47.64, NA) | 0.59 (0.46, 0.74) |  |
| Grade | |  |  |  |  | 0.0901s |
|  | 1 | 60 | 14 | NA (NA, NA) | 0.8 (0.71, 0.92) |  |
|  | 2 | 411 | 98 | NA (NA, NA) | 0.81 (0.77, 0.85) |  |
|  | 3 | 2809 | 778 | NA (NA, NA) | 0.75 (0.74, 0.77) |  |
| Receptor | |  |  |  |  | 0.9727 |
|  | Triple Negative | 2699 | 734 | NA (NA, NA) | 0.76 (0.75, 0.78) |  |
|  | HR+/Her2- | 801 | 211 | NA (NA, NA) | 0.76 (0.73, 0.8) |  |
|  | Her2+ | 141 | 42 | NA (NA, NA) | 0.75 (0.68, 0.82) |  |
| Surgery type | |  |  |  |  | < 0.0001 |
|  | Lumpectomy | 2049 | 357 | NA (NA, NA) | 0.84 (0.82, 0.86) |  |
|  | Mastectomy | 2601 | 813 | NA (NA, NA) | 0.69 (0.67, 0.71) |  |
| Radiation | |  |  |  |  | < 0.0001 |
|  | None | 1682 | 466 | NA (NA, NA) | 0.74 (0.71, 0.76) |  |
|  | Breast/chest wall | 2846 | 649 | NA (NA, NA) | 0.78 (0.76, 0.8) |  |
| Clinical staging | |  |  |  |  |  |
| cT | |  |  |  |  | < 0.0001 |
|  | 1 | 1451 | 205 | NA (NA, NA) | 0.87 (0.85, 0.89) |  |
|  | 2 | 2289 | 547 | NA (NA, NA) | 0.78 (0.76, 0.79) |  |
|  | 3 | 621 | 272 | 80.1 (64.39, NA) | 0.55 (0.51, 0.59) |  |
|  | 4 | 268 | 142 | 44.94 (37.75, 72.48) | 0.44 (0.38, 0.51) |  |
| cN | |  |  |  |  | < 0.0001 |
|  | 0 | 3820 | 852 | NA (NA, NA) | 0.79 (0.78, 0.8) |  |
|  | 1 | 623 | 221 | NA (NA, NA) | 0.65 (0.61, 0.69) |  |
|  | 2 | 111 | 61 | 42.15 (29.54, 97.02) | 0.42 (0.33, 0.53) |  |
|  | 3 | 62 | 32 | 52.57 (42.97, NA) | 0.41 (0.29, 0.58) |  |
| Clinical Stage | |  |  |  |  | < 0.0001 |
|  | 1 | 1386 | 187 | NA (NA, NA) | 0.88 (0.86, 0.9) |  |
|  | 2 | 2605 | 673 | NA (NA, NA) | 0.76 (0.74, 0.77) |  |
|  | 3 | 658 | 310 | 54.8 (44.94, 72.48) | 0.48 (0.44, 0.53) |  |
| Pathologic staging | |  |  |  |  |  |
| pT | |  |  |  |  | < 0.0001 |
|  | 0 | 46 | 6 | NA (NA, NA) | 0.87 (0.78, 0.97) |  |
|  | is | 14 | 2 | NA (NA, NA) | 0.85 (0.68, 1) |  |
|  | 1 | 1436 | 189 | NA (NA, NA) | 0.88 (0.86, 0.9) |  |
|  | 2 | 2002 | 498 | NA (NA, NA) | 0.78 (0.76, 0.8) |  |
|  | 3 | 579 | 260 | 89.17 (68.07, NA) | 0.55 (0.51, 0.59) |  |
|  | 4 | 185 | 123 | 26.51 (21.75, 40.64) | 0.32 (0.26, 0.41) |  |
| pN | |  |  |  |  | < 0.0001 |
|  | 0 | 3342 | 686 | NA (NA, NA) | 0.81 (0.8, 0.83) |  |
|  | 1 | 630 | 250 | 121.76 (106.78, NA) | 0.63 (0.59, 0.67) |  |
|  | 2 | 150 | 78 | 50.2 (39.92, NA) | 0.44 (0.36, 0.54) |  |
|  | 3 | 53 | 33 | 38.83 (19.84, 81.22) | 0.39 (0.27, 0.55) |  |
| Pathologic Stage | |  |  |  |  | < 0.0001 |
|  | 0 | 39 | 3 | NA (NA, NA) | 0.92 (0.84, 1) |  |
|  | 1 | 1334 | 156 | NA (NA, NA) | 0.9 (0.88, 0.92) |  |
|  | 2 | 2357 | 622 | NA (NA, NA) | 0.76 (0.74, 0.78) |  |
|  | 3 | 521 | 292 | 42.97 (36.9, 52.27) | 0.43 (0.38, 0.48) |  |
| Pathologic complete response | |  |  |  |  | 0.0348 |
|  | Yes | 85 | 15 | NA (NA, NA) | 0.83 (0.75, 0.92) |  |
|  | No | 4565 | 1155 | NA (NA, NA) | 0.76 (0.74, 0.77) |  |

HR, hormone receptor
NACT, neoadjuvant chemotherapy

* Overall survival data were unavailable for 486 patients. Survival analysis was conducted on 4650 patients with available overall survival time and event data.
